# Epidemiology of marine turtle fibropapillomatosis and tumour-associated chelonid alphaherpesvirus 5 (ChHV5; Scutavirus chelonidalpha5) in North-Western Mexico: a scoping review implementing the one health approach

**DOI:** 10.1007/s11259-024-10429-6

**Published:** 2024-06-26

**Authors:** Joelly Espinoza, Alonzo Alfaro-Núñez, Carlos Cedillo-Peláez, Helena Fernández-Sanz, Agnese Mancini, Alan A. Zavala-Norzagaray, Cesar Paul Ley-Quiñonez, Erika Santacruz López, Miguel Angel Garcia-Bereguiain, A. Alonso Aguirre, Eduardo Reséndiz

**Affiliations:** 1 Posgrado en Ciencias Marinas y Costeras (CIMACO), Universidad Autónoma de Baja California Sur (UABCS), Carretera al Sur Km 5.5., Apartado Postal 19-B, 23080 La Paz, Baja California Sur Mexico; 2 Health assessments in sea turtles from B.C.S, La Paz, 23085 Baja California Sur México; 3 Department of Clinical Biochemistry, Naestved Hospital, Ringstedgade 57a, Naestved, 4700 Denmark; 4 Section for Evolutionary Genomics, GLOBE Institute, University of Copenhagen, Øster Farimagsgade 5, Copenhagen K, 1353 Denmark; 5 Laboratorio de Inmunología experimental, Instituto Nacional de Pediatría, Insurgentes Cuicuilco, Av. Insurgentes Sur 3700, Coyoacán, Ciudad de México, 04530 Mexico; 6 Grupo Tortuguero de las Californias A.C, La Paz, 23098 Baja California Sur Mexico; 7 Instituto Politécnico Nacional, Centro Interdisciplinario de Investigación para el Desarrollo Integral Regional (IPN-CIIDIR), Mexico City, Mexico; 8 Grupo tortuguero de Bahía de los Ángeles, Bahía de los ángeles, 22980 Baja California Mexico; 9 One Health Research Group, Universidad de Las Américas, Quito, Ecuador; 10 Department of Fish, Wildlife, and Conservation Biology, Warner College of Natural Resources, Colorado State University, Fort Collins, CO USA; 11 Departamento académico de Ciencia Animal y Conservación del Hábitat, Universidad Autónoma de Baja California Sur (UABCS), Carretera al Sur KM 5.5., Apartado Postal 19-B, La Paz, 23080 Baja California Sur México; 12 Asociación Mexicana de Veterinarios de Tortugas A.C, Xalapa, 91050 Veracruz México

**Keywords:** Baja California, Chelonid fibropapilloma-associated herpesvirus, CFPHV, Chelonid herpesvirus 5, Epidemiology, Marine turtle fibropapillomatosis

## Abstract

Fibropapillomatosis (FP) - tumour-associated chelonid alphaherpesvirus 5 (ChHV5; Scutavirus chelonidalpha5) - is a disease that affect marine turtles around the world, and characterized by the formation of cutaneous tumours that can appear anywhere on the body. We carried out a thorough literature search (from 1990 to 2024) in the feeding sites of North-western Mexico, a region that hosts important habitats for feeding, development, and reproduction for five of the seven existing sea turtle species. We found 18 reports recording a total of 32 cases of FP and/or ChHV5/Scutavirus chelonidalpha5 in coastal and insular areas of North-western Mexico. Baja California Sur resulted with the highest number of cases (75%). While the first case of ChHV5/Scutavirus chelonidalpha5 infection was reported in 2004, the presence of FP tumours was reported in 2014 and became more frequent between 2019 and 2024. The affected species were black, *Chelonia mydas* (50%), olive ridley, *Lepidochelys olivacea* (46.8%) and loggerhead turtles, *Caretta caretta* (3.2%). Tumours occurred mainly in anterior flippers (46.1%) and neck (22.5%), and most had a nodular and verrucous appearance with a rough surface. In the study region, there is a potential sign of the emergence of the ChHV5/Scutavirus chelonidalpha5 infections and FP disease during the last 20 years, with a rapid increase during the last 10 years. As long as infections by ChHV5/Scutavirus chelonidalpha5 and the prevalence of the FP disease may be potentially influenced by anthropogenic activities, a One Health approach is needed to understand and improve sea turtles’ health.

**Supplementary Information:**

The online version contains supplementary material available at 10.1007/s11259-024-10429-6.

## Introduction

The Ocean’s health is intrinsically influenced by climate, temperature, topography, currents and by the health of its living organisms, including humans and marine turtles. As such, to understand and preserve a good health for this multivariable system, it is necessary to adopt a transdisciplinary and collaborative approach such as the One Health framework (Prata et al. 2022). Interest is given to the current trend of a rise of infectious diseases in marine organisms (Tracy et al. 2019) with special consideration to the potential consequences of human-driven activities that can alter natural ecosystems (Manes et al. 2023). Marine turtle fibropapillomatosis (FP) is a neoplastic disease characterized by the development of single or multiple epithelial or fibro-epithelial tumours identified as papilloma, fibropapilloma or fibroma that can occur with internal tumours, in advanced stages of the disease (Work et al. 2004). FP was initially reported in free-ranging green turtles (*Chelonia mydas*) in Florida, United States in 1937 (Smith and Coates 1938), and years later, in 1980, FP cases were reported in captive green turtles (Jacobson et al. 1989). Since 1990, the prevalence of the disease increased alarmingly in different areas of Florida and Hawaii (Herbst 1994) but in Hawaii declined over time for no known clear reasons (Chaloupka et al. 2009). Currently, FP has been reported in all species of sea turtles (Huerta et al. 2000; Alfaro-Núñez et al. 2014) in practically all the world’s oceans, with incidence that vary depending on the species populations, time, and space (Alfaro-Núñez et al. 2016; Tagliolatto et al. 2016; Jones et al. 2022). Although the disease can have fatal consequences, regression of tumours has also been reported (Bennett et al. 1999; Guimarães et al. 2013; Kelley et al. 2022). The cause(s) of the disease remain partially undetermined; however, a multifactorial aetiology has been proposed closely related to environmental conditions (Herbst and Klein 1995; Arthur et al. 2008; Dos Santos et al. 2010; Van Houtan et al. 2014; Alfaro-Núñez et al. 2014; Manes et al. 2022; Victor et al. 2022). FP has been associated with both, enveloped and non-enveloped viruses including a papilloma-like virus, a retrovirus, and the *Chelonid Herpesvirus 5* (ChHV5/Scutavirus chelonidalpha5), a double-stranded DNA virus belonging to the subfamily Alphaherpesvirinae, genus Scutavirus (Herbst et al. 1995; Lu et al. 2000; Aguirre and Lutz 2004; Ackermann et al. 2012). However, only ChHV5/Scutavirus chelonidalpha5 has systematically been detected in FP tumours and in clinically healthy animals across multiple studies over the years proven to be the most likely etiological agent for FP (Alfaro-Núñez et al. 2016), as well as the additional in vitro replication in cultures of green turtle skin cells achieved (Work et al. 2017). Nevertheless, confirmation of ChHV5/Scutavirus chelonidalpha5 as the primary causal agent of FP tumours remains inconclusive as defined by the four Koch’s postulates that establish relationships to identify the causative agent of a disease (Alfaro-Núñez et al. 2016) attribute to the difficulty and fruitless attempts over decades to cultivate and isolate the virus.

Affected turtles usually present from one to multiple fibroepithelial tumours (FPs) and in some cases, internal tumours of different sizes. Morphologically, FPs can vary in anatomical distribution, size, appearance, consistency, and coloration (Herbst et al. 1995). Lesion sizes can vary from 0.1 to ≥ 30 cm in diameter (Work and Balazs 1999), frequently presenting necrosis and ulceration in larger tumours (Jacobson et al. 1989). Tumour consistency may be firm or smooth (Page-Karjian et al. 2014; Monezi et al. 2016), and a wide range of colorations have been reported (Gámez et al. 2009). Turtles severely affected with FP are often emaciated, weak, anaemic, and immunosuppressed (Aguirre et al. 1999; Work et al. 2001); also, they tend to have high parasite loads (Work et al. 2005) and an increased risk of developing secondary infections (Work et al. 2003). Large external and internal tumours can cause blindness, buoyancy problems, kidney failure, gastrointestinal occlusion, and in some instances, death (Herbst et al. 1995). Additionally, it has been reported that FP can decrease growth rates in afflicted marine turtles (Chaloupka and Balazs 2005), and it can have negative effects both at the individual and at the population level (Stacy 2016). Macro and microscopic evaluation of FP have been suggested to provide insight into how the disease affects sea turtles in different regions of the world (Herbst et al. 1999). For example, oropharyngeal tumours are common in Hawaiian green turtles, but less common on Florida and Brazil (Aguirre et al. 2002; Page-Karjian et al. 2014; Rossi et al. 2016). Additionally, it has been reported in captive green turtles that individuals with corneal tumours have the worse survival prognosis, while turtles with flat plaque tumours are more likely to survive than turtles with warty and nodular tumours (Page-Karjian et al. 2014).

In the eastern Pacific (EP) region, FP and ChHV5/Scutavirus chelonidalpha5 have been reported in black (or East Pacific green turtles) (*Chelonia mydas*), olive ridley (*Lepidochelys olivacea*) and leatherback (*Dermochelys coriacea*) turtles either at feeding or nesting sites of USA, Mexico, Nicaragua, Costa Rica, Ecuador, and Chile (McDonald and Dutton 1990; Aguirre et al. 1999; Huerta et al. 2000; Quackenbush et al. 2001; Brenes et al. 2013; Alfaro-Núñez et al. 2014; Reséndiz et al. 2015, 2016; Chaves et al. 2017; Cárdenas et al. 2019; Álvarez-Varas et al. 2019; Espinoza et al. 2020). In Mexico, the reports of FP-like lesions began in the 1990s in olive ridley and Kemp´s (*Lepidochelys kempii*) turtles on mass nesting beaches in Oaxaca (Pacific) and Tamaulipas (Gulf of Mexico), respectively (Barragan and Sarti 1994; Aguirre 1998). The first confirmed case of FP was in a nesting leatherback turtle in Michoacán (Pacific) in 1997 (Huerta et al. 2000). Since then, FP reports have been sporadic in black and olive ridley turtles in the states of Oaxaca, Colima, Sinaloa, Baja California Sur (BCS) (Gámez et al. 2009; Reséndiz et al. 2015, 2016; Mejía-Radillo et al. 2019), as well as green turtles in the states of Veracruz, Yucatán, and Quintana Roo (Maldonado-Gasca and Zapata-Rosales 2007; Suárez-Domínguez et al. 2020; Muñoz et al. 2022). On the other hand, ChHV5/Scutavirus chelonidalpha5 molecular records are exclusively reported in the Pacific region (Quackenbush et al. 2001; Mejía-Radillo et al. 2019; Espinoza et al. 2020; Reséndiz et al. 2021). Although the study of FP is limited in the EP region, reports of turtles affected by FP have increased in several Pacific sub-regions during the last decade, including north-western (NW) Mexico (Reséndiz et al. 2022).

Five species of sea turtles inhabit the coastal waters of the NW Mexico Pacific Ocean and the Gulf of California (GC) at different stages of their life cycles. The black turtle feeds in its coastal lagoons, where they remain for years until reaching sexual maturity (Seminoff et al. 2002; Senko et al. 2010). Olive ridley and hawksbill (*Eretmochelys imbricata*) turtles feed and nest in the coastal waters and beaches of the region (Zavala et al. 2008; Cuevas et al. 2010); while loggerhead (*Caretta caretta*) turtles develop and feed in this region (Seminoff et al. 2004). Leatherback turtles nest occasionally on the southern beaches of the Baja California Peninsula (BCP) (Seminoff and Dutton 2007). Even though FP has been extensively studied in regions like Hawaii and Florida, this disease has been poorly studied in other regions, including the temperate latitudes of the EP region, where several sea turtle species converge (Álvarez-Varas et al. 2019; Buenrostro-Silva et al. 2022). Therefore, it is important to understand how FP and associated ChHV5/Scutavirus chelonidalpha5 develop and manifest in distain latitudinal marine turtle populations, as different manifestations of the disease have been reported according to unique population segments linked to ecological and genetic characteristics (Herbst et al. 1999; Work et al. 2020; Álvarez-Varas et al. 2021). There are other alternative theories suggesting a potential latitudinal gradient associated to the development of different viral diseases (Zambrano-Mila et al. 2020), which may be worth it to be evaluated in future research.

The objectives of this study are as follow: a-) to synthesize the current knowledge on the occurrence of FP and ChHV5/Scutavirus chelonidalpha5 cases in marine turtles in the feeding sites and nesting beaches of NW Mexico, b-) to describe the spaciotemporal distribution of the cases and the characteristics of the external lesions, and c-) to describe the main findings that suggest the presence of a herpesvirus (ChHV5/Scutavirus chelonidalpha5). Additionally, every objective was examined in combination of the possible impact that human-driven activities carried out in NW Mexico could have on the development of this disease, addressing the One Health approach. Thereby, this will lead to a better understanding of FP in the region with the implementation of proper protocols for the recognition of FP as it spreads across sea turtles inhabiting NW Mexico. Finally, the information generated in our study will support policy makers on the importance of marine wildlife diseases and their holistic use as indicators for coastal environmental quality in NW Mexico.

## Materials and methods

### Study area

**Fig. 1:**
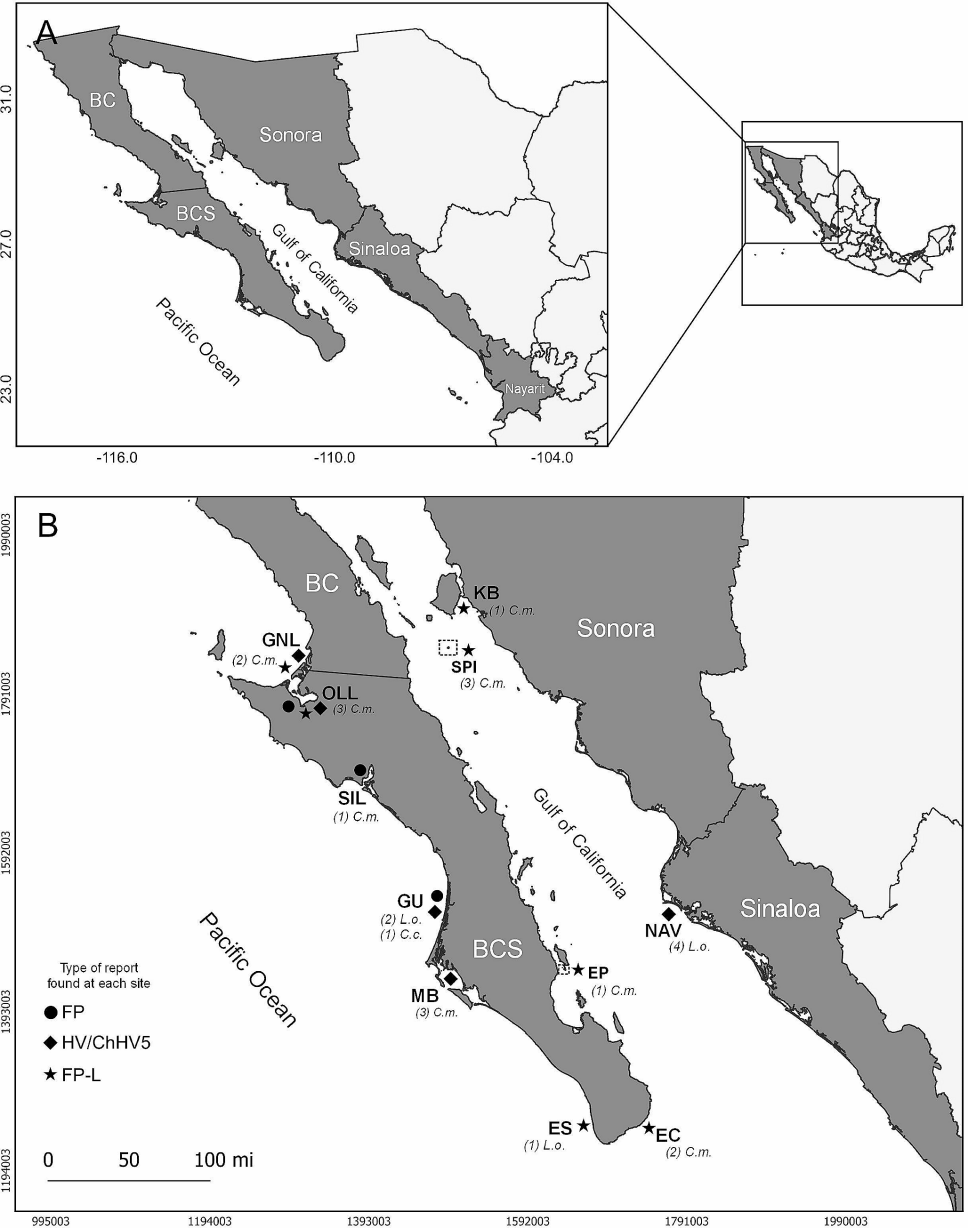
North-western region map. A: Map of North-western Mexico. BC: Baja California; BCS: Baja California Sur. B: Sites and species where FP and ChHV5/Scutavirus chelonidalpha5 occurred in the period 2004–2024 in BCS (GNL: Guerrero Negro Lagoon; LOL: Ojo de Liebre Lagoon; SIL: San Ignacio Lagoon; GU: Gulf of Ulloa; MB: Magdalena Bay; ES: El Suspiro beach; EC: El Cardón; EP: El Pardito); Sonora (KB: Kino Bay; SPI: San Pedro Mártir Island); and Sinaloa (NAV: Navachiste). Islands (EP and SPI) are shown inside of a dotted lines box. Symbols represent the types of cases reported for each site; Circle: Site with confirmed cases of FP; Diamond: Site with confirmed cases of HV or ChHV5/Scutavirus chelonidalpha5; Star: Site with unconfirmed cases of FP (FP-L). The number of individuals by species reported per site is shown in parentheses. C.m.: *Chelonia mydas*; L.o.: *Lepidochelys olivacea*; C.c.: *Caretta caretta*

NW Mexico (Fig. 1A) includes the BCP, with the states of BC and BCS, and the states of Sonora, Sinaloa, and Nayarit on the mainland. The BCP is bordered to the west by the Pacific Ocean and to the east by the GC, while Sonora, Sinaloa and Nayarit form the eastern coast from the GC. On the Pacific coast different masses of water converge, including the California Current, which together with the prevailing coastal winds from the north produce a coastal upwelling of nutrients, making it a highly productive area (Zaytsev et al. 2003). The GC is the only evaporation basin in the Pacific Ocean and is characterized by its high biological productivity and by its different hydrographic characteristics, among them, its circulation and surface temperature, which is predominantly seasonal (Mardones et al. 1999).

### Data compilation

This study was conducted according to the Arksey and O’Malley’s framework (Arksey and O’Malley 2005). Likewise, it is reported following the specifications of the PRISMA-ScR (Preferred Reporting Items for Systematic reviews and Meta-Analyses extension for Scoping Reviews) checklist (Tricco et al. 2018). For the realization of this review, the following questions were identified: (i) What is the spatio-temporal distribution of FP and ChHV5/Scutavirus chelonidalpha5 in NW Mexico? (ii) What are the macroscopic characteristics of FP lesions in sea turtles from NW Mexico? (iii) What are the diagnostic techniques that have been used to diagnose the disease and the associated etiologic agent? For this purpose, published and unpublished records reporting FP and ChHV5/Scutavirus chelonidalpha5 in sea turtles captured in the states that conform the NW Mexico region were reviewed.

A search was carried out in June 2021, March 2023 and finally January 2024. Literature search was conducted through databases including PubMed, Scopus, SciELO, and Google Scholar. The search period spanned from 1990, when the disease was first reported in Mexico, to January 2024. The search strategy included a series of specific keywords that were selected based on the objectives of the study. Keywords were combined with the Boolean operators AND/OR, and included fibropapillomatosis, fibropapilloma, Chelonid herpesvirus 5, herpesvirus, CFPHV, ChHV5/Scutavirus chelonidalpha5, sea turtles, *Chelonia mydas*, *Lepidochelys olivacea*, *Caretta caretta*, *Eretmochelys imbricata*, black turtle, green turtle, olive ridley, loggerhead, hawksbill, North-western Mexico, Baja California, Baja California Sur, Sonora, Sinaloa, Nayarit, Mexican Pacific, Gulf of California, Eastern Pacific, both in English and Spanish (see Additional file 1 for the specific search terms and search equation used); Based on the requirements of the consulted databases, the necessary modifications were made in the search strategy. Additionally, a manual search that consisted of scanning the reference lists of eligible articles and other relevant articles was performed. While, in the case of unpublished reports, a search was made (by the reviewer A.M.) on the records of the sea turtle capture-mark-recapture program performed in NW Mexico by Grupo Tortuguero de las Californias (GTC) A.C.

All the records identified in databases and additional resources were exported in a Microsoft Excel 2019 spreadsheet in order to continue applying the selection criteria. Once duplicate articles and articles in a language other than English and Spanish were excluded, articles were screened by title and abstract, those that appeared to meet inclusion criteria were screened in full text for eligibility, and based on the inclusion criteria were included or excluded. The inclusion criteria addressed confirmed (microscopic and molecular studies that reports findings compatible with FP and/or herpesvirus infection) and non-confirmed (the presence of external tumours) cases of FP and/or herpesvirus infection citing NW Mexico region as study area. Studies that did not mention the study area or the number of individuals affected with FP/herpesvirus were excluded.

The presence and frequency of FP and ChHV5/Scutavirus chelonidalpha5 infections were collected through the documentation of (a) publications in peer-reviewed journals (b) internal technical reports, (c) unpublished reports and (d) thesis. Full texts of the selected records were analysed to extract the following data: authors, publication year, species, date of capture (mm/dd/yy) of each individual sea turtle with a precision of at least year, geographical region of capture (the area was tabulated, specifying the location where the event occurred), morphometric measurements of the captured turtles and characteristics of external tumours, detection technique used (if performed) and main diagnostic findings reported by the original authors of the different studies. In addition, an attempt was made to include photographs of the evaluated reports where possible. Curved Carapace Length (CCL; cm) and weight (kg) were recorded (Bolten 1999), life-stage (Márquez 1990; Ishihara and Kamezaki 2011; Hart et al. 2014) and sex were compiled when was possible (Wyneken 2001). Descriptive statistics were performed to identify the number together with the percentages of confirmed and non-confirmed cases of FP/herpesvirus, year of publication of the analysed reports, species, and capture sites of each individual sea turtle.

### Characterization of tumours

Based on photographs and data obtained in the report search described above, tumours were macroscopically categorized according to the size of the lesion base (diameter in cm), anatomical distribution (ocular region, head, neck, shoulders, axillary region, anterior and posterior extremities, carapace, plastron, inguinal and cloacal region), appearance, (verrucous, nodular, plaque, cauliflower-like or mixed), surface (smooth, rough, papillomatoses or mixed) and coloration (Herbst 1994; Rossi et al. 2016). The size was measured using ImageJ software (Schneider et al. 2012). The severity degree was assigned according to the protocol proposed by Work and Balazs (1999). The presence of external parasites in the tumours and ulceration was also considered (Jacobson et al. 1989). Descriptive statistics were performed to identify the number together with the percentages of severity degree of FP, anatomical distribution of FP lesions, as well as the appearance and surface of the lesions.

## Results

### Study selection

**Fig. 2:**
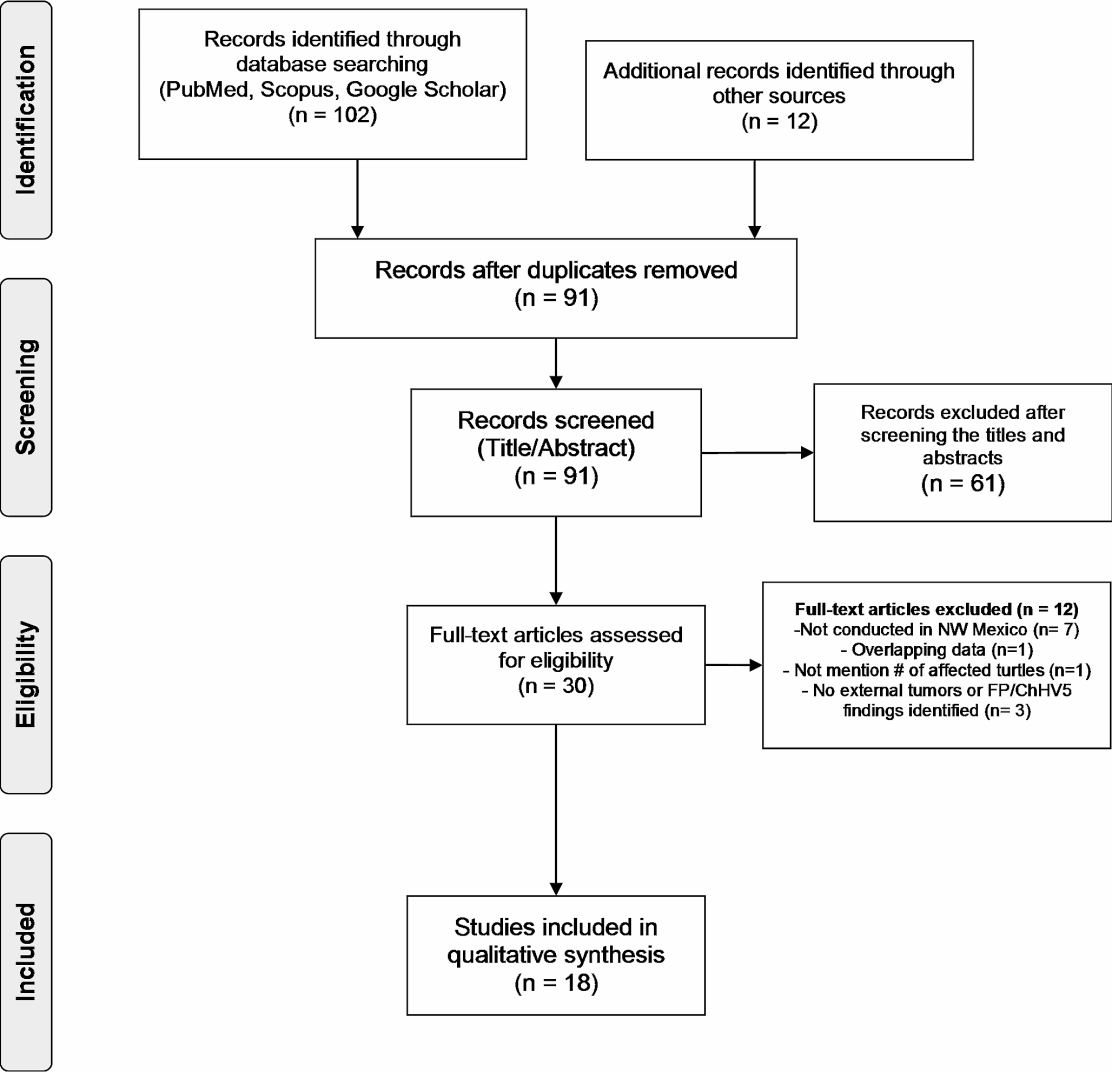
PRISMA flow diagram of the screening and selection process in the scoping review

From the database search, 102 studies were identified: 16 from PubMed, 20 from SCOPUS, 66 from Scholar Google, and 0 from SciELO. Aditionally, 10 reports were identified through the Grupo Tortuguero de las Californias AC (GTC) database and two reports through the manual search of the reference lists. In total, 114 studies were identified. After removing duplicates, 91 articles were identified for title and abstract screening. A total of 61 studies were excluded in the title and abstract screening because the topic was not related, or the studies were in a language other than English or Spanish, or they were not conducted in NW Mexico region (studies that do not mention the study site in the title or abstract, were analyzed in the full text), and 30 full text studies were assessed for eligibility. After the full-text assessed, 12 studies were excluded and 18 were included for data extraction and analysis. The article screening process is shown in Fig. 2.

### Published and non-published FP/herpesvirus reports in NW Mexico

Based on the search criteria, 32 cases of affected sea turtles, extracted from 18 published or unpublished studies were identified for NW Mexico. Fourteen confirmed cases (14/32, 43.7%) of either FP or herpesvirus infection were collected from seven published reports: five peer-reviewed journals publications, one technical report and a thesis (Castelán 2004; Cordero-Tapia and Reséndiz 2014; Reséndiz et al. 2016, 2019b, 2021; Mejía-Radillo et al. 2019; Espinoza et al. 2020). Whereas the remaining 18 suspected cases of FP (18/32, 56.3%) were collected from the GTC database and one technical report (Vega Hernández 2023). In 2014 the first confirmed FP cases were reported in olive ridley and loggerhead turtles from BCS, NW Mexico (Cordero-Tapia and Reséndiz 2014). However, herpesvirus infection by ChHV5/Scutavirus chelonidalpha5 was originally reported in clinically healthy black turtles since 2004, also in BCS (Castelán 2004). No herpesvirus nor confirmed FP records exist prior 2004 and 2014, respectively. However, a report of suspected FP case in a black turtle captured in 2010 was found in the GTC database, although screening analyses were never performed. In 14 (43.7%) sea turtles from the seven reports, FP and/or herpesvirus infection were confirmed using histopathology (HP), Transmission Electron Microscopy (TEM), and molecular methods (Table 1). In the remaining 18 cases (56.3%) the detection of FP-like lesions was done through a visual inspection, observing the lesions as solitary or multiple raised masses. However, for the later no sampling or diagnostic tests were carried out to confirm these cases (Table 1).

**Table 1:** Confirmed and non-confirmed cases of FP and/or herpesvirus infection in marine turtles from North-Western Mexico

Turtle No.	Species^a^	Date (mm/dd/yy)	Site^b^	Size (cm), mass (kg)	Age class^c^	Sex^d^	Diagnostic test and findings^e^	Reference
Confirmed FP or Herpesvirus infection cases								
1	Cm	02/16/02	MB	na	na	na	PCR: Positive to Herpesvirus	(Castelán 2004)
2	Cm	03/25/02	MB	na	na	na	PCR: Positive to Herpesvirus	(Castelán 2004)
3	Cm	08/06/02	MB	na	na	na	PCR: Positive to Herpesvirus	(Castelán 2004)
4	Lo	07/23/13	GU	54.0, 30	A	M	HP: Fibroepithelial proliferation and cytopathic damage	(Cordero-Tapia and Reséndiz 2014)
5	Cc	04/02/14	GU	78.0, na	J	U	HP: Fibroepithelial proliferation and cytopathic damage	(Cordero-Tapia and Reséndiz 2014)
6	Cm	05/07/10	SIL	50.3, 16	J	U	HP and TEM: Fibroepithelial proliferation and presence of viral particles	(Reséndiz et al. 2016)
7	Lo	2016	NAV	59.7 to 62, 25 to 38	A	M	PCR: Positive to ChHV5/Scutavirus chelonidalpha5	(Mejía-Radillo et al. 2019)
8	Lo		NAV		A	F		
9	Lo		NAV		A	M		
10	Lo		NAV		A	M		
11	Lo	10/21/16	GU	70.5, 35	A	F	HP and PCR: Fibroepithelial proliferation / positive to ChHV5/Scutavirus chelonidalpha5	(Reséndiz et al. 2019b; Espinoza et al. 2020)
12	Cm	11/08/16	LOL	75.6, 37	J	U	HP: Fibroepithelial proliferation	(Reséndiz et al. 2019b)
13	Cm	09/27/17	GNL	76.3, 46	J	U	PCR: Positive to ChHV5/Scutavirus chelonidalpha5	(Espinoza et al. 2020)
14	Cm	07/18/19	LOL	76.9, 43	J	U	HP and PCR: Fibroepithelial proliferation / positive to ChHV5/Scutavirus chelonidalpha5	(Reséndiz et al. 2021)
Non-confirmed FP cases								
15	Cm	09/10/10	LOL	96.5, 100	A	F	ME: Fibropapilloma-like tumours	GTC^f^
16	Cm	09/10/16	KB	65.5, 30	J	U	ME: Fibropapilloma-like tumours	GTC
17	Cm	11/13/19	EP	57.8, 22	J	U	ME: Fibropapilloma-like tumours	GTC
18	Cm	12/18/19	GNL	55.4, 18	J	U	ME: Fibropapilloma-like tumours	GTC
18	Cm	06/21/20	SPI	65.0, 31	J	U	ME: Fibropapilloma-like tumours	GTC
20	Cm	07/26/20	SPI	71.0, 39	J	U	ME: Fibropapilloma-like tumours	GTC
21	Lo	09/15/20	ES	na	A	F	ME: Fibropapilloma-like tumours	GTC
22	Cm	10/11/20	SPI	64.0, 25	J	U	ME: Fibropapilloma-like tumours	GTC
23	Cm	12/14/20	EC	71.4, 39	J	U	ME: Fibropapilloma-like tumours	GTC
24	Cm	12/14/20	EC	59.3, 24	J	U	ME: Fibropapilloma-like tumours	GTC
25	Lo	2022	ES	60 to 70, na	A	F	ME: Fibropapilloma-like tumours	(Vega Hernández 2023)
26	Lo		ES		A	F	ME: Fibropapilloma-like tumours	(Vega Hernández 2023)
27	Lo		ES		A	F	ME: Fibropapilloma-like tumours	(Vega Hernández 2023)
28	Lo		ES		A	F	ME: Fibropapilloma-like tumours	(Vega Hernández 2023)
29	Lo		ES		A	F	ME: Fibropapilloma-like tumours	(Vega Hernández 2023)
30	Lo	2022	ES	na, na	A	F	ME: Fibropapilloma-like tumours	(Vega Hernández 2023)
31	Lo		ES		A	F	ME: Fibropapilloma-like tumours	(Vega Hernández 2023)
32	Lo		ES		A	F	ME: Fibropapilloma-like tumours	(Vega Hernández 2023)

^a^Species: Cm: *Chelonia mydas*; Lo: *Lepidochelys olivacea*; Cc: *Caretta caretta*

^b^Site: *MB* Magdalena Bay; *SIL* San Ignacio Lagoon; *GU* Gulf of Ulloa; *NAV* Navachiste; *LOL* Ojo de Liebre Lagoon; *KB* Kino Bay; *EP* El Pardito; *GNL* Guerrero Negro Lagoon; *SPI* San Pedro Mártir Island; *ES* El Suspiro beach; *EC* El Cardón

^c^Age class: *J* Juvenil; *A* Adult

^d^Sex: *F* Female; *M* Male; *U* Unidentified

^e^Diagnostic test and findings: *HP* Histopathology; *TEM* Transmission Electron Microscopy; *PCR* Polymerase Chain Reaction; *ME* Macroscopic evaluation; *na* Information not available

^f^Reference: *GTC* Official database of Grupo Tortuguero de las Californias A.C. (GTC), including annual visual reports of FP-like lesion, but non-confirmed

**Fig. 3:**
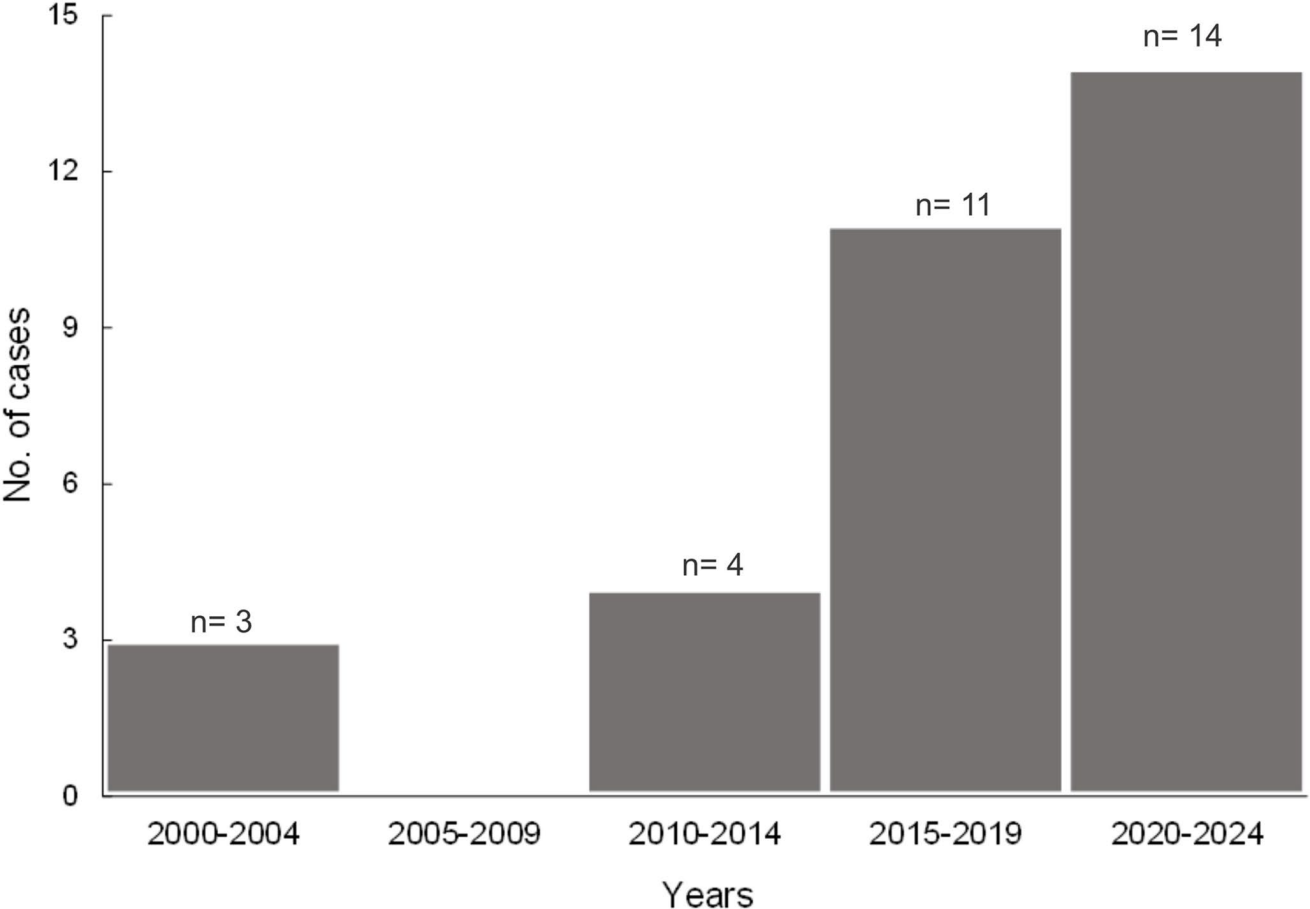
Cases of FP/ChHV5/Scutavirus chelonidalpha5 in sea turtles from North-western Mexico from 2000 to 2024. Diagram showing the number of reports of FP/ChHV5/Scutavirus chelonidalpha5 from 2000 to 2024, with an increase in cases during the last decade. The years refer to the dates of capture of the individuals. We speculate that one of the potential reasons for this increase may be associated with the increment in the monitoring effort and the additional recognition and understanding of the disease in the region

Three affected turtles were captured in 2002 (9.3%), two in 2010 (6.2%), one in 2013 (3.2%), one in 2014 (3.2%), seven in 2016 (21.8%), one in 2017 (3.2%), three in 2019 (9.3%), six in 2020 (18.7%) and eight in 2022 (25%) (Fig. 3). Regarding the capture sites of the affected individuals, twenty-four (24/32, 75%) cases were reported in BCS state including three cases each in Magdalena Bay (MB), Ojo de Liebre Lagoon (LOL), and Gulf of Ulloa (GU) (9.3% per site), two cases each in El Cardón (EC) and Guerrero Negro Lagoon (GNL) (6.2% per site), one case each in San Ignacio Lagoon (SIL) and El Pardito (EP) (3.2% per site) and nine in El Suspiro beach (ES) (28.1%), a solitary nesting beach for olive ridley turtles; four cases of turtles (4/32, 12.5%) with lesions were reported in Sonora state including three in San Pedro Island (SPI) (9.3%), and one in Kino Bay (KB) (3.2%). Additionally, four cases (4/32, 12.5%) were reported in Sinaloa state, specifically in Navachiste (NAV) (Table 1; Fig. 1B). No FP/ChHV5/Scutavirus chelonidalpha5 reports were found in the states of Baja California (BC) or Nayarit.

Black turtles represented 50% of the total of reported individuals (16/32) and included 12 immature individuals, one adult female and three unknown adults. The morphometric information of the black turtles (*n* = 3) derived from Castelán (2004) was not available. Olive ridley turtles represented 46.8% of cases (15/32), including eleven adult females and four adult males. Finally, a unique immature loggerhead turtle represented 3.2% (1/32) of cases (Table 1).

### Macroscopic analysis of FP

Regarding the degree of severity of FP, 17 turtles were classified with tumour score 1 or mildly affected, seven turtles with tumour score 2 or moderately affected, while the black turtle no. 15 was classified as tumour score 3 or severely affected (Table 2). It was not possible to calculate the degree of severity of FP or characterize the FP-like lesions in three individuals (individuals 26, 27 and 32) because the number of tumours per individual or their photographic record were not available (Vega Hernández 2023). The remaining individuals did not present clinical signs of FP (individuals 1–3 and 5).

**Table 2:** Macroscopic characterization of tumours in marine turtles from North-Western Mexico

Turtle No. (Species)	Anatomic distribution	Tumour No.^a^	Size (cm)	Appearence^b^ Surface^c^	Color^d^	Score^e^
4 (Lo)	Proximal dorsal region of anterior flippers	4 right − 5 left	0.5 to 2	NV, R	P, G, Y	1
6 (Cm)	Lateral canthus of each eye	1 right − 1 left	3	NV, R	P, G, W	2
	Ventral surface of both hind flippers	7 right − 6 left	0.2 to 2	NV, R	P, G, W	
7 (Lo)	Lateral canthus of the right eye	1	< 3	V, R	P	1
8 (Lo)	Ventral region of the right anterior flipper	2	1 to 3	V, R	P, Y, W	1
9 (Lo)	Dorso-proximal region of the right anterior flipper	1	< 3	N, R	P, G, B	1
10 (Lo)	Dorsal region of the neck	1	< 3	P, R	G, B	1
11 (Lo)	Lateral canthus of the right eye	1	1	N, R	P	1
	Proximal ventral surface of right anterior flipper	2	2 to 4	V, R	P, Y	
12 (Cm)	Ventral base of right anterior flipper	1	3	N, R	P, W	1
13 (Cm)	Lateral canthus of the eye	1	na	na	na	1
14 (Cm)	Right mid-lateral neck region	1	1.5	P, R	G, Gn, Br	1
15 (Cm)	Extended in neck, axillary regions and soft tissue areas	Multiple fused tumours	˃10	CL, M	P, Y, Pp, G	3
16 (Cm)	Cranial proximal region of the right anterior flipper	3	1 to 3	V, P	P, Pp, Y, W	1
17 (Cm)	Lateral canthus of the right eye	1	0.6	N, S	P, B	2
	Right lateral neck region	1	2	N, P	P, Y, Pp, G	
	Dorsal proximal region of the anterior flipper	7	1 to 3	N, P	P, Y, Pp, G	
18 (Cm)	Proximal dorsal-caudal region of right anterior flipper	1	1.5	N, P	G, Gn, Y	1
19 (Cm)	Proximal dorsal-caudal region of left anterior flipper	1	< 1	N, S	P, G, Pp	1
20 (Cm)	Central ventral region of the neck	1	0.5	P, R	P, G	1
	Cranial proximal region of left anterior flipper	1	1.3	V, R	Br, Y	
	Lateral canthus of left eye	1	1.5	N, S	P, G, Pp	
21 (Lo)	Left lateral neck region	2	1	V, R	P	1
	Left shoulder	1	0.5	V, R	P	
22 (Cm)	Dorsal proximal region of right anterior flipper	5	2 to 4	NV, R	P, B, G, Gn	2
	Lower region of the right shoulder	1	1	N, R	P, Y	
	Left axillary region	1	0.8	V, R	P, G, W	
	Cranioventral proximal region of the left anterior flipper	8	0.5 to 1.5	N, R	P, G, W	
23 (Cm)	Medial canthus of the left eye	1	0.5	V, R	P	1
24 (Cm)	Dorsal central region of the neck	1	6.3	V, P	B, G, Br, W	2
	Left shoulder-flipper junction	1	3	V, P	B, G, Br, W	
25 (Lo)	Lateral neck region	4 right − 1 left	1 to 3	na	na	1
26 (Lo)	Both shoulders	Multiple lesions	1	V, na	B	na
27 (Lo)	Left anterior flipper	Multiple lesions	2 to 4	N, na	P, G	na
28 (Lo)	Right shoulder, below the third marginal scute	1	6	CL, na	P, G	2
29 (Lo)	Left shoulder, below the third marginal scute	1	11	N, na	P, G	2
30 (Lo)	Left shoulder	1	1	N, na	na	1
31 (Lo)	Lateral regions of the neck	4 right − 4 left	2	na	na	2
32 (Lo)	Neck, shoulders, and both anterior flippers	Multiple lesions	3	V, na	G, B	na

Cm: *Chelonia mydas*; Lo: *Lepidochelys olivacea*

^a^Number of tumours per anatomical region

^b^Appareance: *V* Verrucous; *N* Nodular; *NV* Nodular and verrucose; *P* Plaque; *CL*: Cauliflower-like

^c^Surface: *R* Rough; *S* Smooth; *P* Papillomatous; *M* Mixed

^d^Coloration: *P* Pinkish; *W* Witish; *Y* Yellow; *Gn* Green; *Pp* Purple; *Br* Brown; *G* Gray; *B* Black

^e^Tumour score category: 1: Slightly affected; 2: Moderately affected; 3: Very affected; na: Information not available

**Fig. 4:**
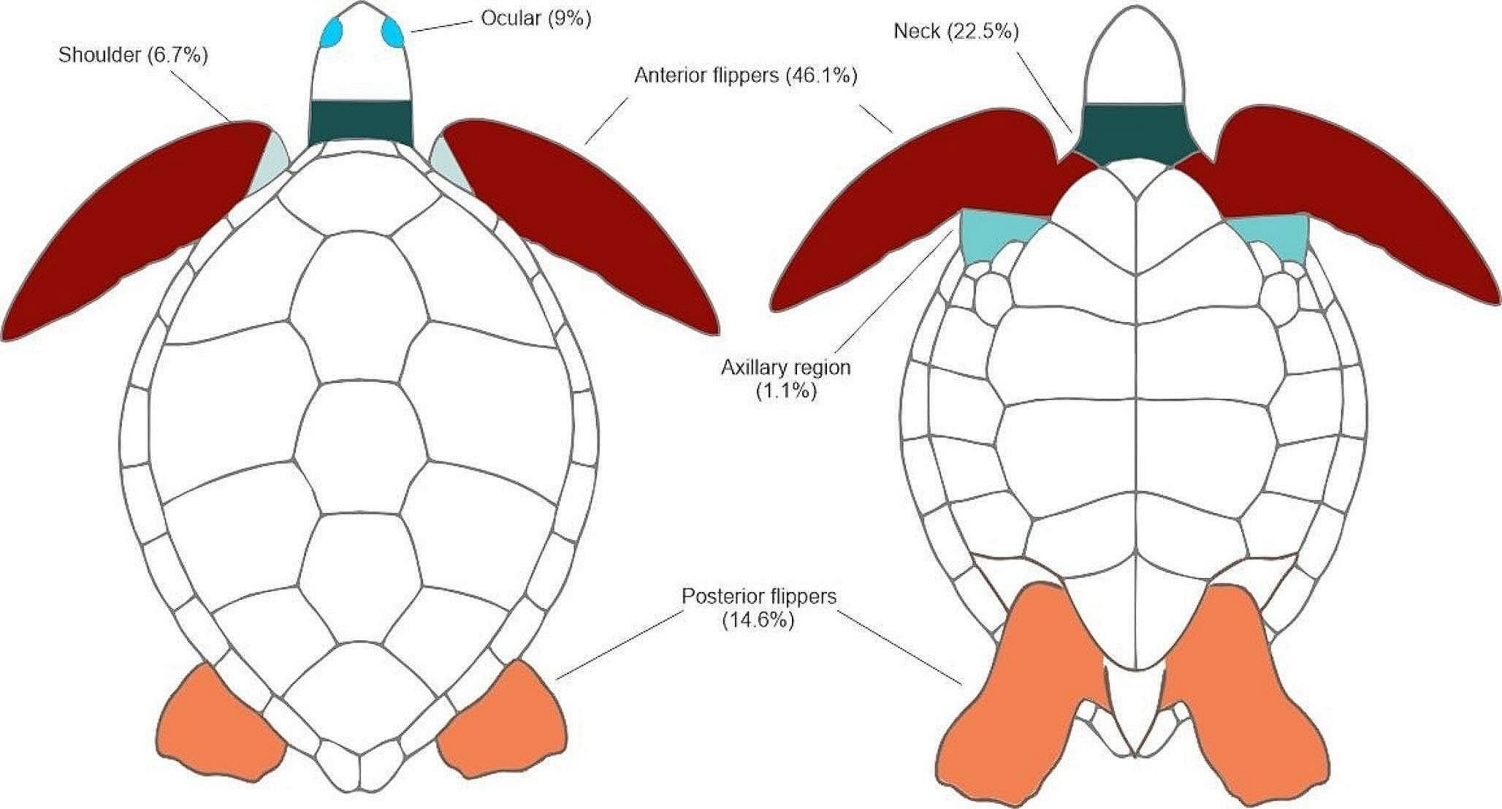
Anatomical distribution of tumours. The percentages refer to the number of individual tumours evaluated (*n* = 89)

Based on the availability of reported data, it was possible to quantify 89 lesions from 24 turtles (Table 2; Fig. 4). The lesions ranged from 0.2 to 11 cm in diameter, whereas the number of tumours per individual ranged from one to fifteen. The 85.4% (76/89) of the tumours were in the anterior portion of the body of the turtles. The anterior flippers (*n* = 41 or 46.1%) and the neck (*n* = 20 or 22.5%) were the most affected followed by posterior flippers (*n* = 13 or 14.6%), eyes (*n* = 8 or 9%), shoulders (*n* = 6 or 6.7%) and axillary regions (*n* = 1 or 1.1%), see Fig. 4.

**Fig. 5:**
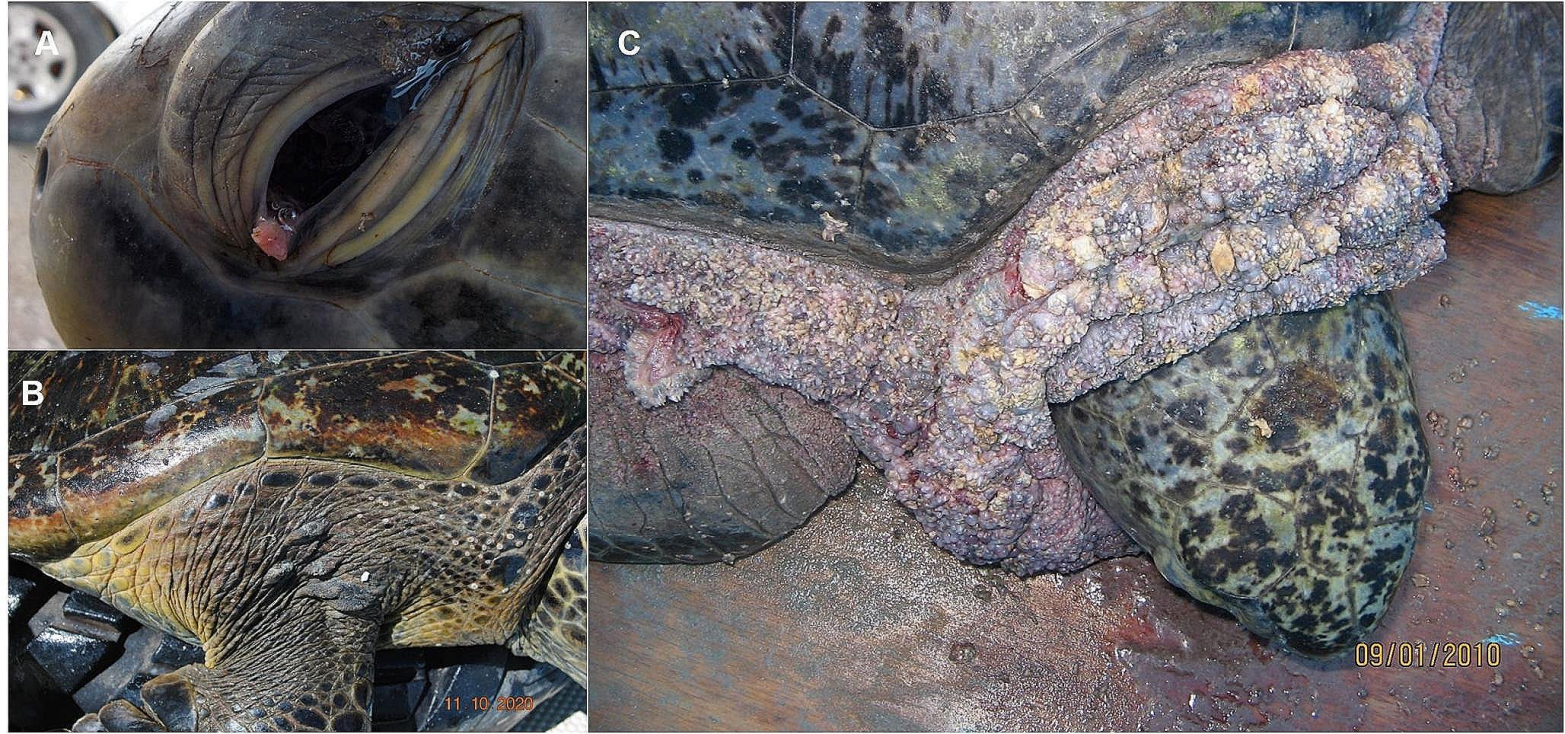
Black showing suggestive signs of FP. Differences in the anatomical distribution, size, location, appearance, and coloration of the tumours can be observed between individuals. **A** Individual 23 showing a pinkish FP-like tumours in the medial canthus of the left eye. **B** Individual 22 showing FP-like lesions in the dorsal proximal region of right anterior flipper, with a mixed appearance and rugose surface. **C** Individual 15, with presence of FP-like tumours extended and merged in neck, shoulder and the proximal region of anterior flippers, a wide range of appearance, coloration and surface are shown. The photographs were courtesy of: Grupo Tortuguero de las Californias, A.C. and Health assessments in sea turtles from Baja California Sur (HASTBCS).

Moreover, it was possible to extract the frequency corresponding to the macroscopic appearance of 75 lesions from 21 individuals. The appearance of the tumours was an up-mixture (nodular/verrucous) in 29 tumours (38.7%), 26 tumours (34.7%) were nodular, 16 (21.3%) were verrucous, three tumours (4. %) had a plaque-like appearance, and one was reported with a cauliflower-like appearance (1.3%). It was possible to retrieve the surface characteristics of 72 tumours from 18 specimens, and it was mainly rough (*n* = 55 or 76.4%), followed by tumours with papillomatous (*n* = 14 or 19.4%) and smooth (*n* = 3 or 4.2%) surface. The coloration of the tumours varied, from pale and intense pink, whitish and yellow, to darker colorations such as green, purple, brown, grey, and black (Fig. 5). A captured adult black turtle reported in LOL (turtle no. 15) presented multiple coalescent and ulcerated cauliflower-like tumours larger than 10 cm distributed in the neck, axillary and inguinal regions as well as soft areas. In this case it was not possible neither quantify nor measure the tumours due to the severe degree of fusion presented (Fig. 5C).

### Findings of FP and herpesvirus infection cases

FP was confirmed by HP in three turtle individuals reported from BCS: two black (individuals 6 and 12, Table 1) from SIL and LOL and one olive ridley (individual 4) from GU (Cordero-Tapia and Reséndiz 2014; Reséndiz et al. 2016, 2019b). Additionally, one loggerhead (individual 5) that did not present visible tumours but histopathological changes consistent with FP were reported in normal skin (Cordero-Tapia and Reséndiz 2014). On the other hand, ChHV5/Scutavirus chelonidalpha5 infection was reported in four olive ridley turtles (individuals 7 to 10) with visible tumours from Sinaloa and in the unaffected skin from a black turtle (individual 13) that presented an ocular tumour from GNL, while three clinically healthy black turtles (individuals 1 to 3) from MB were PCR-positive to Herpesvirus infection (Castelán 2004; Mejía-Radillo et al. 2019; Espinoza et al. 2020). In addition, there was one olive ridley (individual 11) from GU and one black (individual 14) turtle from LOL that were diagnosed with FP and ChHV5/Scutavirus chelonidalpha5 infection by HP and molecular analysis of the tumours, respectively (Espinoza et al. 2020; Reséndiz et al. 2021). In the rest of the individuals (15 to 32) the analysis of the lesions was only at the macroscopic level (Vega Hernández 2023). Tumours analysed by HP were classified as fibropapilloma since they presented proliferation of the epidermis and dermis, whereas the histopathological changes reported in the normal skin from the loggerhead turtle resembled an early presentation of FP. Orthokeratosis, hyperkeratosis, acanthosis, dermal papillary differentiation were some of the lesions reported in all the study cases (Cordero-Tapia and Reséndiz 2014; Reséndiz et al. 2016, 2019b, 2021). Finding related to viral infections such as ballooning degeneration were also reported in all cases, while the presence of Intranuclear Inclusion Bodies (IIBs) in the keratinocytes was reported in an olive ridley with FP (individual 4) and in the clinically healthy loggerhead (individual 5) (Cordero-Tapia and Reséndiz 2014). In addition, icosahedral viral particles similar in morphology, size, and site of replication to those reported in herpesvirus infection were reported by TEM in an olive ridley with FP captured in SIL, individual 6 (Reséndiz et al. 2016).

Regarding the herpesvirus infection study, Castelán (2004) reported herpesvirus variants that coincided in 44% with green turtle herpesvirus (GTHV) (former name for ChHV5/Scutavirus chelonidalpha5) variants from Hawaii and Florida. More recently, the authors reported EP variants of ChHV5/Scutavirus chelonidalpha5 in all samples analysed (Mejía-Radillo et al. 2019; Espinoza et al. 2020; Reséndiz et al. 2021).

## Discussion

### Historical epidemiology of FP and ChHV5/Scutavirus chelonidalpha5 in the region

This study summarizes the available evidence on the occurrence of FP cases and ChHV5/Scutavirus chelonidalpha5 infection in multiple species of sea turtles inhabiting NW Mexico. In addition, through the development of our study, it was observed an important empty gap of knowledge regarding the study of the FP disease in the region. Historically, only 32 marine turtles with FP and/or herpesvirus infection have been reported in the NW region of Mexico, including 22 published cases from either peer-reviewed journals, internal reports, or thesis, and 10 unpublished cases from the GTC database records. Confirmed cases occurred only in the states of BCS and Sinaloa, while in Sonora were only speculative. On the other hand, there were no reports of the disease or ChHV5/Scutavirus chelonidalpha5 in BC or Nayarit, which seems to be associated to the lack of monitoring of the disease in the areas, rather than the apparent absence of the disease. Since, only one study carried out in Bahia de los Angeles, an important feeding site located on the coast of the GC of BC state, in which five clinically healthy black turtle skin samples were negative for ChHV5/Scutavirus chelonidalpha5, was found (Espinoza et al. 2020). Following this logic, one potential scenario to explain the variation in FP cases across the years may suggest that the detection of herpesvirus in the research sites has rather been random and circumstantial, and not as a direct result of a standardize monitoring strategy (Jones et al. 2022). Likewise, the increase in FP appearance in recent years may be attributed to a better recognition and understanding of the disease than in previous years (Fig. 3); and/or because in some feeding areas the monitoring effort has been greater than in the past (Jones et al. 2022).

Considering the historical data, it is likely that ChHV5/Scutavirus chelonidalpha5 has been passively spreading (possibly in latency or lethargy stage) for decades in the feeding sites of NW Mexico, with or without the sporadic manifestation of FP (Alfaro-Núñez et al. 2016) but not efforts were consistently made to detect the presence of the viral disease. In this sense, infected turtles in latently stage that later on began to develop visible tumours, could be related to unknown environmental and/or host immunity factors (Alfaro-Núñez et al. 2016; Lawrance et al. 2018; Yetsko et al. 2020, 2021). Non-infectious factors appear to play an important role in the pathogenesis of virus-associated neoplasia diseases (such as FP) and are often related to pollution caused by human activities, which may generate a change (or imbalance) in host-pathogen interactions via immunosuppression, resulting in a potential increased disease frequency and/or severity with serious consequences for the animal, environmental, and human health (Bossart 2011; Bossart and Duignan 2019; Hamede et a. 2020; Baines et al. 2021). In the case of FP, it has been suggested that some environmental variables such as sea surface temperature or salinity may be correlated with the high incidence of FP (Manes et al. 2022; Vanstreels et al. 2023) across species populations worldwide. In addition, a higher occurrence of FP has been reported in highly urbanized areas with eutrophication, process that produces a decrease in the macroalgae diversity consumed by green turtles (Dos Santos et al. 2010; Van Houtan et al. 2014; Victor et al. 2022). The macroalgae consumed by green turtles in impacted environments are mainly invasive, and compared to native macroalgae, have a greater potential to sequester high Nitrogen (N) contents in the form of arginine, an amino acid that has been reported as a tumour promoter (Dos Santos et al. 2010; Van Houtan et al. 2014). Other studies report a relationship with biotoxins such as Lyngyatoxin A, brevetoxins and okadaic acid, produced by some toxic dinoflagellates that proliferate on the algae and seagrasses that green turtles consume (Landsberg et al. 1999; Arthur et al. 2008; Perrault et al. 2017). Hence, the quality of the diet of green turtles (modified by human activities) could be a key cofactor in the development of the disease. Conversely, although no direct relationship has been found between FP and organochlorine pesticides (OCPs), PAHs (Polycyclic Aromatic Hydrocarbons) or heavy metals such as Cu, Fe, As, Cd and Hg, as there has also been proposed that chronic exposure to these compounds could cause immunosuppression and thus play a role in tumour progression, mainly in areas highly impacted by mining and industrial waste (Keller et al. 2014; da Silva et al. 2016; Vilca et al. 2018; Miguel et al. 2022). Therefore, to understand the complex pathogenesis and impact of FP, it is necessary to systematically monitor the turtle’s health together with anthropogenic activities and environmental factors that could potentially influence the spreading and incidence of the infectious disease by applying the One Health approach (Bossart and Duignan 2019) and to continuously incorporate the disciplines that study health at the animal, human and environmental levels. While no studies have been carried out in NW Mexico to evaluate the potential relationship between the quality of the environment or the diet with the expression of FP, studies of the physical and haematological condition of black, olive and loggerhead turtles in some feeding site of BCS (MB, LOL, SIL and GU) report a “good” state of health, without evident alterations to suspect a weakened immune system (Ley-Quiñónez et al. 2017; Reséndiz et al. 2018, 2019a). However, it has been suggested the potential risk that represents the persistent presence of some heavy metals such as Cd, As, Hg, Zn, and Se in the health of black, olive and loggerhead turtles from Sonora (Ley-Quiñónez et al. 2013), Sinaloa (Zavala-Norzagaray et al. 2014; Olimón-Andalón et al. 2021), BC (Presti et al. 1999) and BCS, where the presence of OCPs has also been reported, although at low levels, in the three mentioned species (Gardner et al. 2003, 2006; Kampalath et al. 2006; Ley-Quiñónez et al. 2017). In general, these results reflect the exposure of a wide range of polluting sources in the coastal ecosystems of NW Mexico (Zavala-Norzagaray et al. 2014), which could be suspected as environmental triggers for the FP manifestation in these feeding grounds.

There is the general perception that the NW Mexico is considered a pristine region with low to moderate levels of contamination (Gardner et al. 2003; Kampalath et al. 2006; Páez-Osuna et al. 2017). However, this region has also been positioned as one of the most important agro-industrial regions in Mexico, causing some coastal areas of the region to be heavily impacted by urban discharges, agricultural runoff, and mining (Frías-Espericueta et al. 2014; Macías and García 2021). Moreover, a risk analysis study carried out in Navachiste (a study site with FP and ChHV5/Scutavirus chelonidalpha5 cases) showed that frequent consumption of snappers (*Lutjanus* spp.) could cause carcinogenic effects in humans due to the accumulation of OCPs (Granados-Galván et al. 2015). In the same way, in BCS (MB) and Sonora state, high concentrations of Fe, Cu, Cd and Mg were recorded in macroalgae and seagrasses, which suggests that could be incorporated into the diet of herbivorous animals, such as green turtles, representing a risk to their health (Riosmena-Rodríguez et al. 2010; Mendez-Rodriguez et al. 2021). Hence, the persistence of OCPs and heavy metals through the trophic web could represent a risk not only as a possible FP co-factor, but also for humans who feed on specimens with presence of these contaminants (Senko et al. 2009). On the other hand, eutrophication has been reported along the coast of NW Mexico (Ruiz-Ruiz et al. 2017). For example, in KB, Sonora (a feeding site with FP-like lesions), N concentrations were higher in areas impacted by effluents from shrimp farms than those not impacted (Barraza-Guardado et al. 2014), which could potentially have a negative effect on sea turtles’ quality diet.

Infection with ChHV5/Scutavirus chelonidalpha5 and the manifestation of FP occur mainly in coastal feeding sites such as bays and lagoons with low water exchange (Ene et al. 2005), and the feeding sites of NW Mexico meet these geographical characteristics (Senko et al. 2010). Additionally, this region has rapidly been impacted by anthropogenic activities that seem to be co-factors to the FP development, therefore it is necessary to perform studies with an interdisciplinary approach that evaluate the health of the coastal ecosystem, but also the health of species such as sea turtles (Aguirre and Lutz 2004). As previously stated, sea turtles are widely considered sentinel species that respond to environmental changes in the ecosystem they inhabit, which helps to identify the negative impacts that these changes could have on public and marine ecosystem health (Aguirre and Lutz 2004; Bossart 2011). One suggested strategy to monitor these environmental changes is through FP epidemiology, in other words, sea turtles and coastal ecosystem health is monitored through the spread, pathogenesis and impact of the disease on sea turtles in certain region (Aguirre and Lutz 2004). However, there is no data on the prevalence or incidence of FP/ChHV5/Scutavirus chelonidalpha5 for feeding sites in NW Mexico, as it is shown in this study, the reports are limited to isolated cases. Therefore, it is necessary to implement a systemic disease surveillance program that measures the frequency of FP/ChHV5/Scutavirus chelonidalpha5 in the region, and that will allow to detect changes in the health status of the sea turtle population in the long term.

Following the One Health approach, the suspected increment in the incidence of FP within the feeding sites of NW Mexico (Fig. 3) may serve to alert about possible negative impacts on the health of the coastal ecosystem, and, in turn, of the potential negative effect that it could have on public health (Aguirre and Lutz 2004; Prata et al. 2022). In this way, the necessary strategies can be developed to mitigate and control both the etiological and the non-infectious factors that may drive the appearance and spread of diseases.

The reported cases of FP in black turtles were mainly presented in juveniles individuals, which is consistent with previous studies from other regions where juvenile green turtles are the most affected by FP (Monezi et al. 2016; Suárez-Domínguez et al. 2020; Jones et al. 2022). Moreover, this could also be influenced by the fact that coastal feeding sites in NW Mexico have a higher proportion of juveniles than adults (Koch 2013). On the contrary, all affected olive ridley turtles were adults. Following the same logic, the study feeding area is mainly inhabited by adult and sub-adults’ olive ridley turtles. Furthermore, the majority of cases in this species were represented by nesting females from ES (Zavala et al. 2008). These findings are relevant because there are previous reports of olive ridley turtles with FP on arribada nesting beaches in the EP (Mexico and Costa Rica) but not on the solitary nesting beaches of NW Mexico, considered as one of the most northern distributions of this species (Aguirre 1998; Aguirre et al. 1999; Reséndiz et al. 2015). Additionally, FP and ChHV5/Scutavirus chelonidalpha5 have also been reported in more southern feeding site of the EP in Chile (Álvarez-Varas et al. 2019), which represents that both FP and ChHV5/Scutavirus chelonidalpha5 are present in the full range of distribution of olive ridley turtles in the EP. Studying the impact that it can have on the health of the population is an important topic to study.

### Pathology of the FP tumours

From the data collected in our study, a maximum of 15 tumours per individual were identified, this is low compared to cases reported in Brazil, where up to 129 tumours per turtle have been found (Rossi et al. 2016), but it coincides with reports from Costa Rica (Brenes et al. 2013), where a maximum of 12 tumours per individual was found in nesting olive ridley turtles. The predominance of small tumours is consistent with the findings in other studies from the Mexican Pacific (Gámez et al. 2009) and Gulf of Mexico (Suárez-Domínguez et al. 2020) but differ from those reported in places where the prevalence of FP is high, such as Hawaii and Florida, where it is common that tumours exceed 10 cm in diameter (Work et al. 2004; Farrell et al. 2018). The differences between the sizes of the tumours may be because the turtles in regions like Hawaii and Florida have presence of the disease for a longer time, and as the disease progresses, the lesions increase in size (Farrell et al. 2018). The anatomical distribution of the tumours was predominant in the anterior region of the body, like what is reported in other regions of the EP (Aguirre et al. 1999; Reséndiz et al. 2015), north Pacific (Work et al. 2004) and Gulf of Mexico (Suárez-Domínguez et al. 2020). It has been reported that the first signs of FP are the growth of small lesions in the anterior part of the body, and this could be explained by the fact that there is more soft tissue in the anterior region than in other parts of the body (Work et al. 2004). Two olive ridley and five black turtles had ocular tumours that ranged between 0.5 and 3.0 cm in diameter. Tumours in the ocular region have been reported in other regions of the EP previously (Aguirre et al. 1999; Brenes et al. 2013). This class of tumours is alarming because turtles (individuals in rehab process) with ocular FP have a worse survival prognosis compared to turtles that present FP in other anatomical areas such as the neck or flippers (Page-Karjian et al. 2014), However, these results differ from those reported in free-living individuals (Hirama and Erhart 2007). Most of the tumours had a nodular and verrucous appearance with a rough surface. These findings are in accordance with those reported in green turtles from Veracruz and Colima, Mexico (Gámez et al. 2009; Suárez-Domínguez et al. 2020). One black turtle from BCS was severely affected by FP. Advanced stages can negatively affect the fitness and ability of turtles to feed, see and swim. Moreover, severely affected turtles tend to have a poor immune response, opening the way for opportunistic infections (Work et al. 2001, 2003). It is possible that the differences in the genetic composition of the virus or of the black turtles at these feeding sites may explain the morphological variations and degree of severity of FP observed in this individual (Tagliolatto et al. 2016).

The histopathological changes reported for all cases are characteristic of FP in sea turtles around the world (Herbst et al. 1999; Kang et al. 2008; Brenes et al. 2013; Monezi et al. 2016). Interestingly, histopathological changes typical of FP were also reported in a clinically healthy juvenile loggerhead (individual 5) from BCS (Cordero-Tapia and Reséndiz 2014). In addition, it presented cytopathic changes such as IIBs, which are indicators of viral lytic replication (Kang et al. 2008; Work et al. 2014, 2020). It has been reported that the earliest histopathological lesions of FP are characterized by proliferation of the epidermis and dermis, degenerative changes, and occasionally IIBs (Jacobson et al. 1989; Herbst et al. 1999; Work et al. 2014; Monezi et al. 2016), and this individual met these criteria. Viral particles that matched the characteristics of HV (Herbst et al. 1995) were observed by TEM in tumours of a juvenile black turtle (Reséndiz et al. 2016). These findings could allow us to suggest the theory that different species of sea turtles, both clinically healthy and FP sick, may be releasing viral particles in common shared feeding areas of NW Mexico, and during reproductive migration in the case of adults, thus potentially promoting the spread of the FP associated etiologic agent (Kang et al. 2008; Senko et al. 2010; Work et al. 2014; Monezi et al. 2016). It should be noted that, although cytopathic changes in skin and tumour were reported, molecular or immunohistochemical tests were not performed to confirm the viral agent caused the lesions (Cordero-Tapia and Reséndiz 2014).

To date, NW Mexico hosts the highest number of FP cases reported in black turtles in the EP. Although the coastal feeding sites of NW Mexico meet the geographical characteristics that are reported for the expression of FP (Ene et al. 2005; Senko et al. 2010), it turns out interesting that FP also affected sea turtles in insular areas such as SPI, the most oceanic island in the GC, which is located 64 km from the coast, and that also has a protected status (Amador-Castro et al. 2021). Another unknown is how the ChHV5/Scutavirus chelonidalpha5 spreads within the feeding sites of NW Mexico, although black turtles show a high degree of fidelity in these regions, it has been observed that some individuals can use different feeding sites (Senko et al. 2010). Thus, these individuals could spread the virus through the coastal foraging sites (Work et al. 2014). In contrast, olive ridleys from the EP show mainly pelagic feeding habits with low fidelity to specific foraging habitats (Plotkin 2010), so they could spread the virus widely throughout the EP. Knowing how viral strains are distributed throughout the north-eastern Pacific and the GC, as well as between sea turtle species, will provide an understanding of viral spread and transmission in this region. In addition, as perspective goal, knowing the presence of ChHV5/Scutavirus chelonidalpha5 eDNA in the water or even from the microbiome composition of these coastal feeding sites represents an interesting field of study which could potentially elucidate further knowledge for the transmission and origins of both FP and the virus.

### Limiting factors in the study

Our study also faced some limitations. Firstly, as a general limitation of this type of study, it is possible that the search strategy used did not identify all the studies within the objective of the study. Secondly, very few studies fell within the inclusion criteria, and 56.3% of the cases found were not confirmed by any diagnostic technique, and thus interpretations of the results could be largely speculative. In addition, since diagnostic techniques for ChHV5/Scutavirus chelonidalpha5 have change over time, the results related to the presence of a herpesvirus infection were difficult to interpret as a whole.

## Conclusions

Our scoping review evidences the knowledge gap concerning the occurrence of FP and its associated aetiological agent at feeding sites in NW Mexico. As such, to implement FP as an environmental marker of degradation at coastal feeding sites in NW Mexico, it is first necessary to implement a continuous and systematic FP surveillance system that allows to establish a baseline of disease behaviour as well as to detect acute changes in the occurrence of the disease and the alterations in the environmental FP-associated cofactors. Moreover, given the importance of the region as a developmental and feeding ground for endangered sea turtles, where different species of diverse natal origins congregate, it is necessary to continue with the capture-mark-recapture programs since these programs generate valuable information that can be used to evaluate the impact of FP (Patrício et al. 2016) on the population of sea turtles in NW Mexico. Therefore, we recommend increasing FP monitoring efforts at feeding sites of BCS, Sonora and Sinaloa, as well as establishing a monitoring program in BC and Nayarit states, to train staff teams to recognize the disease, to do the proper management of sick individuals, as well as to collect the necessary samples to carry out the studies required for the detection of FP and its viral etiological agent, following the biosecurity measures to prevent the spread of the disease. In addition, create alliances with academic and government institutions to carry out diagnostic tests. An effective long-time surveillance of FP will allow to identify disease trends and hotspots over time, to create and adapt management plans and conservation strategies by the corresponding health authorities, as well as to use FP as an environmental marker of degradation for the habitats where sea turtles congregate in the NW of Mexico. Likewise, creating alliances with institutions that work on environmental and public health monitoring issues around these feeding sites will help generate comprehensive knowledge from the One Health perspective.

## Supplementary Information

Below is the link to the electronic supplementary material.

Supplementary file1 (DOCX 14 kb) **Table S1**. Keywords used to retrieve scientific publications from the different databases and search equation.

## Data Availability

No datasets were generated or analysed during the current study.
