# Supplementary material for: Epidemiology of marine turtle fibropapillomatosis and tumour-associated chelonid alphaherpesvirus 5 (ChHV5; Scutavirus chelonidalpha5) in North-Western Mexico: a scoping review implementing the one health approach
Source: Vet Res Commun. 2024 Jun 26;48(5):2943–61. doi: 10.1007/s11259-024-10429-6 (PMC11442556; doi:10.1007/s11259-024-10429-6)
Supplement: Supplementary file 1 — Supplementary file1 (DOCX 14 kb) Table S1. Keywords used to retrieve scientific publications from the different databases and search equation. [file 11259_2024_10429_MOESM1_ESM.docx]

**Table S1.** Keywords used to retrieve scientific publications from the different databases and search equation.

| ***Fibropapillomatosis* –**  related keywords | "fibropapillomatosis" OR "fibropapilloma" OR "fibropapillomas" OR "chelonid herpesvirus 5" OR "herpesviridae" OR "herpesviridae" OR "herpesviridae" OR "herpesvirus" OR "alphaherpesvirus" OR "cfphv" OR "chhv5" |
| --- | --- |
| ***Sea turtles* –**  related keywords | "sea turtle" OR "marine turtle" OR "chelonia mydas" OR "lepidochelys olivacea" OR "caretta caretta" OR "eretmochelys imbricata" OR "black turtle" OR "green turtle" OR "olive ridley" OR "loggerhead" OR "loggerheads" OR "hawksbill" OR "hawksbills" |
| ***Northwestern Mexico* –**  related keywords | "northwestern mexico" OR "baja california" OR "baja california sur" OR "baja california peninsula" OR "sonora" OR "sinaloa" OR "nayarit" OR "mexican pacific" OR "eastern pacific" OR "mexico" OR "gulf of california" |
| ***Equation for PubMed Database***  (Fibropapillomatosis AND sea turtles AND Northwestern Mexico AND 1990 to 2024) | ("fibropapillomatosis"[All Fields] OR ("fibropapilloma"[All Fields] OR "fibropapillomas"[All Fields]) OR "chelonid herpesvirus 5"[All Fields] OR "herpesviridae"[MeSH Terms] OR ("herpesviridae"[MeSH Terms] OR "herpesviridae"[All Fields] OR "herpesvirus"[All Fields]) OR "alphaherpesvirus"[All Fields] OR "cfphv"[All Fields] OR "chhv5"[All Fields]) AND ("sea turtle*"[All Fields] OR "marine turtle*"[All Fields] OR "chelonia mydas"[All Fields] OR "lepidochelys olivacea"[All Fields] OR "caretta caretta"[All Fields] OR "eretmochelys imbricata"[All Fields] OR "black turtle*"[All Fields] OR "green turtle*"[All Fields] OR "olive ridley"[All Fields] OR ("loggerhead"[All Fields] OR "loggerheads"[All Fields]) OR ("hawksbill"[All Fields] OR "hawksbills"[All Fields])) AND ("northwestern mexico"[All Fields] OR "baja california"[All Fields] OR "baja california sur"[All Fields] OR "baja california peninsula"[All Fields] OR "sonora"[All Fields] OR "sinaloa"[All Fields] OR "nayarit"[All Fields] OR "mexican pacific"[All Fields] OR "eastern pacific"[All Fields] OR "mexico"[All Fields] OR "gulf of california"[All Fields]) AND 1990/01/01:2024/01/17[Date - Publication] |
